# Prognostic Role of PSMA-Targeted Imaging in Metastatic Castration-Resistant Prostate Cancer: An Overview

**DOI:** 10.3390/biomedicines12102355

**Published:** 2024-10-16

**Authors:** Matteo Caracciolo, Angelo Castello, Massimo Castellani, Mirco Bartolomei, Egesta Lopci

**Affiliations:** 1 Nuclear Medicine Unit, Oncological Medical and Specialists Department, University Hospital of Ferrara, 44124 Ferrara, Italy; matteo.caracciolo@ospfe.it (M.C.); m.bartolomei@ospfe.it (M.B.); 2 Nuclear Medicine Unit, Fondazione IRCCS Ca’ Granda, Ospedale Maggiore Policlinico, 20089 Milan, Italy; angelo.castello@policlinico.mi.it (A.C.);; 3 Nuclear Medicine Unit, IRCCS—Humanitas Research Hospital, Rozzano, Via Manzoni 56, 20089 Milan, Italy

**Keywords:** prostate cancer, PSMA, prostate-specific membrane antigen, PET, prognosis, mCRPC, metastatic castration-resistant prostate cancer

## Abstract

Objectives: Prostate-specific membrane antigen (PSMA) positron emission tomography/computed tomography (PET/CT) has gained a primary role in prostate cancer (PCa) imaging, overcoming conventional imaging and prostate-specific antigen (PSA) serum levels, and has recently emerged as a promising technique for monitoring therapy response in metastatic castration-resistant prostate cancer (mCRPC) patients treated with novel hormonal therapy, taxanes, and radioligand therapy (RLT). In this review, we aim to provide an overview of the most relevant aspects under study and future prospects related to the prognostic role of PSMA PET/CT in mCRPC. Methods: A systematic literature search was performed in the following databases: MEDLINE, PubMed, and EMBASE databases. The study focused exclusively on English-language studies, excluding papers not pertinent to the topic. Results: PSMA PET imaging offers a higher sensitivity and specificity than conventional imaging and provides accurate staging and efficient diagnosis of distant metastases. The data presented herein highlight the usefulness of PET in risk stratification, with a prognostic potential that can have a significant impact on clinical practice. Several prospective trials are ongoing and will shortly provide more evidence supporting the prognostic potential of PET PSMA data in this clinical scenario. Conclusions: Current evidence proves the prognostic role of PSMA PET/CT in different settings, with raising relevance also in the context of mCRPC.

## 1. Introduction

Prostate cancer (PCa) is becoming one of the most prevalent neoplasms worldwide, with 1.3 million diagnoses every year and approximately 2.3 million new cases expected by 2040 [1]. Androgen deprivation therapy (ADT) has become the mainstay of treatment for patients with recurrent or advanced disease [2]. Nevertheless, approximately 10%–20% of patients develop, within 5 years from starting ADT, a further biochemical relapse despite low levels of serum testosterone (≤50 ng/dL), a clinical condition defined as castration-resistant prostate cancer (CRPC) [3]. The Prostate Cancer Working Group 3 (PCWG3) defines this condition when PSA levels increase 25% from the nadir, considering only a rise of at least 2 ng/mL; whereas, according to the European Association of Urology (EAU), two consecutive values of PSA higher than 0.2 ng/mL are indicative of biochemical progression [4,5]. In addition, the diagnosis of metastatic castration-resistant prostate cancer (mCRPC) significantly decreases the overall survival [6].

In this context, docetaxel has been demonstrated as the only effective therapy able to prolong survival in patients with CRPC [7]. Several other drugs have been proposed, such as abiraterone acetate and enzalutamide, known as androgen-signaling-targeted inhibitors, or cabazitaxel, a next-generation taxane, although a clear indication of the best treatment sequence and combination of these agents is still under debate [8,9].

PCa cells are characterized by overexpression of prostate-specific membrane antigen (PSMA), a transmembrane glycoprotein, whose levels are correlated with disease progression, making it a favorable diagnostic and therapeutic tool in mCRPC [10]. In the last years, PSMA positron emission tomography (PET) has gained a primary role in different clinical settings; for example, to guide biopsy, in the initial diagnosis, or in patients eligible for salvage radiotherapy, as well as for recurrence detection, although it is not free from limitations and challenges. False positive and negative findings can occur and should be kept in mind [11,12,13]. Moreover, PSMA PET is emerging as a promising technique for monitoring therapy responses in mCRPC patients treated with novel hormonal therapy, taxanes, and radioligand therapy (RLT) over conventional imaging and prostate-specific antigen (PSA) serum levels [14,15]. Nevertheless, data regarding PSMA PET in patients with mCRPC are still limited, and it is unclear how treatments may have an impact. Indeed, current PCWG3 guidelines still recommend computed tomography (CT) and bone scans for treatment response evaluation. On the other hand, it is true that in parallel with the decrease in total tumor burden, PSMA PET/CT also shows the appearance of new lesions, leaving the oncologists in a clinical dilemma [4,16,17].

In this review, our aim was to examine the prognostic role of PSMA PET in mCRPC patients with a complete overview of the most relevant aspects under study and of the future prospects.

## 2. Methods

This comprehensive review involved an extensive literature search, encompassing studies published within 31 July 2024. The search was executed using the following string: ((((castration-resistant prostate cancer[Text Word]) OR (CRPC[Text Word])) AND ((PSMA[Text Word]) OR (prostate-specific membrane antigen[Text Word]))) AND ((positron emission tomography[Text Word]) OR (PET[Text Word]))) AND ((((prognosis[Text Word]) OR (survival[Text Word])) OR (response[Text Word])) OR (response assessment[Text Word])). A systematic literature search was performed in the following databases: MEDLINE, PubMed, and EMBASE databases. The study focused exclusively on English-language studies, excluding papers not pertinent to the topic.

## 3. Prognostic Role under Antiandrogen Drugs

As above-mentioned, ADT is the standard therapy for advanced and metastatic PCa, although acquired castration resistance inevitably occurs within a couple of years. Abiraterone acetate (AA) has shown the capability to prolong OS in mCRPC patients, but with a great heterogeneity and widely variable response duration in different mCRPC patients. Therefore, several predictors of response to AA have been proposed, such as neutrophil to lymphocyte ratio or circulating adrenal androgen levels, even though it is still premature for their introduction into clinical practice [18,19].

Recently, Ke et al. [20] explored whether semi-quantitative parameters extracted from [^68^Ga]-PSMA-11 PET/CT could predict PSA response and prognosis in 106 mCRPC cases receiving AA. As a matter of fact, low values of both PSMA tumor volume (PSMA-TV) and tumor-to-liver ratio (TLR) were predictors of biochemical response, and they were associated with better clinical outcomes. Therefore, the combination of ADT duration, PSMA-TV, and TLR distinguished responders from non-responders in mCRPC well.

In a small cohort of only six patients, Markowski et al. [21] analyzed the utility of [^18^F]-DCFPyL PET/CT in determining clinical response to bipolar androgen therapy. Half of the patients had progression on PET/CT imaging, while stable disease or response was detected on conventional imaging, suggesting [^18^F]-DCFPyL PET/CT as a valid tool for early progression assessment.

On the other hand, Karyağar et al. [22] evaluated the predicting role of parameters extracted from PSMA in 34 mCRPC patients receiving enzalutamide. In those with PSA response, PSMA-TV values were significantly lower than non-responders (78.37 ± 80.99 cm^3^ vs. 451.58 ± 734.61 cm^3^; *p =* 0.028), whereas max and mean standardized uptake values (SUVmax and SUVmean) and total lesion (PSMA-TL) were not significantly different between the two groups. Similarly, Plouznikof et al. [23] investigated the association between PSMA expression, by PET/CT, and the response to either enzalutamide or AA in mCRPC. PSMA PET response was thus perfectly associated with conventional response criteria, and no flare phenomenon was detected. Conversely, Shagera et al. [24] investigated the role of PSMA PET for response assessment and outcome prediction in 30 mCRPC patients treated with androgen receptor pathways inhibitors (ARPIs). The results showed a significant correlation between PSA-based and PSMA-based responses, with the latter evaluated according to the EAU/European Association of Nuclear Medicine (EANM) and Response Evaluation Criteria in PSMA PET/CT (RECIP) 1.0 criteria (rho = 0.79 and 0.66, respectively). Moreover, PSMA-responders showed a longer median OS than PSMA non-responders (54 vs. 22 mo) according to both EAU/EANM and RECIP 1.0 criteria.

## 4. Prognostic Role during Chemotherapy

Chemotherapy with docetaxel is the most active treatment for mCRPC patients. As an antimicrotubular agent, docetaxel has a high cytotoxic effect, arresting the mitotic cycle and inducing cell apoptosis [25]. Similarly, cabazitaxel is a novel taxane-based chemotherapy agent, adopted to suppress docetaxel resistance. A historical trial (i.e., TROPIC) supported the efficacy of cabacitaxel plus prednisone compared to mitoxantrone plus prednisone in 755 patients with mCRPC. In this study, the median values of OS and PFS was significantly longer in patients treated with cabazitaxel compared to those with docetaxel (15.1 vs. 12.7 months for OS and 2.8 vs. 1.4 months for PFS, both *p* < 0.0001). Likewise, objective Response Evaluation Criteria in Solid Tumors (RECIST) and PSA response rates were significantly higher in the cabacitaxel group (14.4% vs. 4.4%, *p* < 0.005; 39.2% vs. 17.8%, *p* < 0.0002, respectively) [26,27].

Pre-clinical studies have demonstrated that the expression of PSMA in human PCa cell lines was not affected by docetaxel, while it caused a decrease in androgen receptor and PSA levels. Hence, these pieces of evidence suggest that PSMA PET distribution could be a useful tool for response assessment [28].

Various factors like neoplasm biology, the presence of visceral metastases, or tumor burden influence the outcome of these patients. In fact, from the CHAARTED study, it emerged that only patients with high-volume disease, defined as ≥4 bone lesions with at least one outside pelvis and spine or visceral metastases, had a benefit from docetaxel [29]. Data analysis indicated that approximately 50% of mCRPC patients could be either no responders to docetaxel or develop resistance [30]. Therefore, it is important to identify those patients who can really benefit from this type of therapy. In this context, it has been reported that PSMA-based parameters may have a role in the stratification of patients into low- and high-volume metastatic disease [31]. For example, Has Simsek et al. aimed to investigate the role of PSMA-derived tumor burden metrics as predictors of docetaxel efficacy in a cohort of 52 mCRPC patients. According to ROC analysis, a cut-off value of 107 cm^3^ for PSMA-TV (AUC = 0.838) and 103 cm^3^ for PSMA-TV (AUC = 0.821) predicted biochemical response with 75% sensitivity and 80% specificity, respectively. Furthermore, elevated PSMA-TV and lactate dehydrogenase (LDH) values were associated with a higher risk of docetaxel failure. Finally, the abovementioned volumetric parameters, as well as age and LDH, were significantly associated with shorter survival [32].

In addition, Shagera et al. [33] investigated the role of PSMA PET in 32 patients receiving a median of six cycles of cabazitaxel. Biomarkers, such as PSA, LDH, and alkaline phosphatase, were recorded and correlated with t PSMA-TV for each patient. Of note, subjects with low tumor volume presented longer PFS and OS than those with high volume, with a median PFS of 21 vs. 12 weeks (*p =* 0.017) and a median OS of 24 vs. 8.5 months (*p =* 0.002), respectively. Furthermore, PSMA-TV remained an independent predictor of OS (*p =* 0.016) on the multivariate analysis, showing its potential as a prognostic biomarker in mCRPC patients treated with cabazitaxel. Likewise, in a larger retrospective study including 54 mCRPC patients who underwent PSMA PET before docetaxel or enzalutamide/abiraterone, Telli et al. [34] demonstrated a moderate positive correlation between pre-treatment PSA levels and both PSMA-TV (rho = 0.582, *p =* 0.004) and PSMA-TL (rho = 0.564, *p =* 0.007). Moreover, age > 70 years (*p =* 0.02), higher PSA (*p =* 0.01), lack of PSA response to first-line treatment (*p =* 0.03), higher number of bone lesions (*p =* 0.02), and higher PSMA-TV (*p =* 0.007) were significantly associated with an increased risk of mortality. In the ROC analysis, the cut-off values for PSMA-TV, PSMA-TL, and PSA levels to predict mortality were 40.1, 289.4, and 24.2, respectively. In particular, patients with low values of PSMA-TV and PSMA-TL, as well as those with PSA below 24.2, had a longer OS. Lastly, the multivariate analysis highlighted PSMA-TV (HR: 1003, 95% CI 1001–1004, *p =* 0.001) and the PSA response (HR: 2.241, 95% CI 1189–4222, *p =* 0.01) as independent predictors of OS. These results suggested that PSMA PET parameters, in particular PSMA-TV, could be useful tools to assess tumor burden and predict long-term survivor in patients with mCRPC.

## 5. PSMA-Targeted Radioligand Therapy

Despite the therapeutic possibilities previously described, mCRPC may further progress. In this scenario, radioligand therapy (RLT) represents a promising option that has been recently approved by the European Medicines Agency and the US Food and Drug Administration [35].

In the same way as the systemic therapies analyzed, PSA values and conventional response criteria are commonly used to evaluate response to RLT. However, quantitative molecular imaging parameters derived from PSMA PET appear to be more efficient, as already found with fluorodeoxyglucose (FDG) in other types of cancer [36,37]. In this regard, the recent REALITY trial evaluated a novel volumetric parameter, the so-called total lesion PSMA (TLP), computed as the sum of volume x SUVmean for all lesions, to assess treatment response to RLT in 102 patients [38]. By using a threshold of −30%/+30%, they obtained significant differences between the three categories: partial response (PR) vs. progressive disease (PD) (*p =* 0.001), PR vs. stable disease (SD) (*p =* 0.001), and SD vs. PD (*p =* 0.018); whereas, common biomarkers did not achieve statistical significance. Yet, in the study by Rosar et al. [39], the possible use of TLP as a predictor of response to RLT has been studied. Of note, early change in TLP has been shown to be an independent predictor of OS (*p =* 0.002), outperforming biochemical response assessment (*p =* 0.515).

Moreover, Kind et al. [40] monitored 70 patients with both gallium and fluoride PSMA PET; in particular, they investigated the prognostic role of PSMA_TV50_, a volumetric parameter obtained using a 50% threshold of SUVmax. In total, 33 patients underwent [^68^Ga]-PSMA and 37 [^18^F]-PSMA PET/CT at baseline and after 2–4 cycles, analyzing the change in PSMA_TV50_ (i.e., ∆PSMA_TV50_) in correlation with OS. Higher PSMA_TV50_ and ∆PSMA_TV50_ were significantly associated with a shorter OS for both PSMA and PET, demonstrating a potential prognostic value.

**Figure 1:**
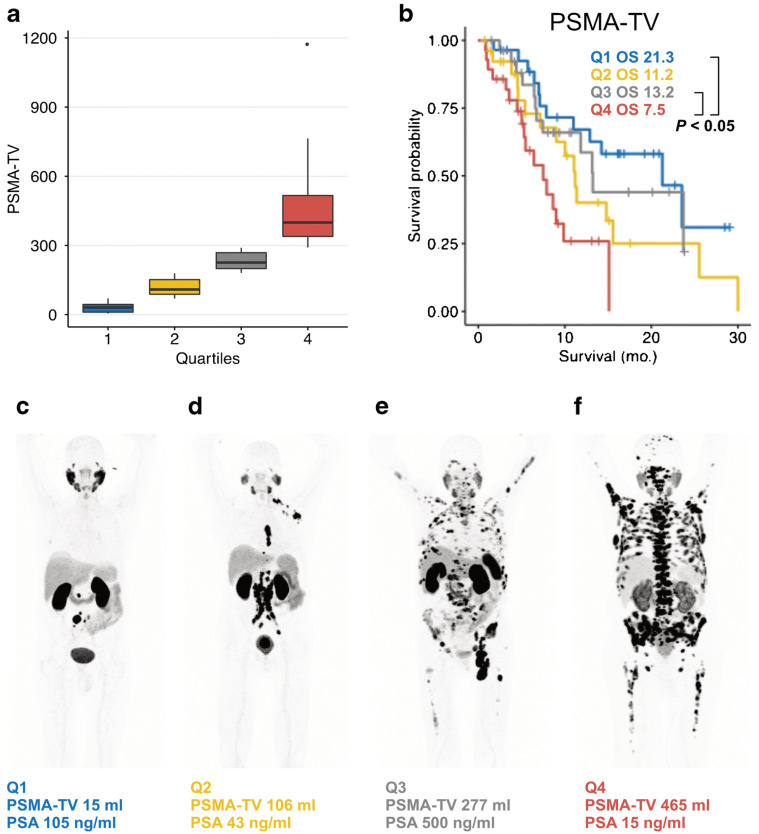
In the present multipanel image, we provide the correlation between PSMA tumor volume (PSMA-TV) expressed in quartiles and overall survival, expressed in months (mo.). Panel (**a**) depicts the boxplots of the PSMA-TV measured in *ml* and the corresponding quartiles 1 to 4. Panel (**b**) provides separate overall survival curves related to each PSMA-TV quartile. Panels (**c**–**f**) provide some exemplary patients corresponding to the different quartiles together with blood levels of prostate-specific antigen (PSA). Reproduced from Seifert, R et al. [37], published under a Creative Commons Attribution 4.0 International License. To view a copy of this license, visit: http://creativecommons.org/licenses/by/4.0/ (accessed on 19 September 2024).

Another aspect of total tumor volume was evaluated by Seifert et al. [41] in a cohort of 33 patients undergoing PSMA PET prior to therapy and after two cycles (Figure 1). They calculated the average PSMA expression (expressed by the mean SUVmax of all of the lesions), PSMA-TV, and total lesion quotient (TLQ = PSMA-TV/ SUVmean). The PSMA-TV value at baseline was a prognostic factors of OS (HR = 1.618 95% CI: 1.117–2.343, *p =* 0.011). On the other hand, the PSMA-TV response became significantly associated with OS only if patients with low mean SUVmax at the baseline were excluded (HR = 0.29; 95% CI: 0.108–0.782; *p =* 0.015). The same group analyzed PSMA-TV, TLQ, and total lesion uptake (PSMA-TLU) in 110 patients treated with [^177^Lu]-PSMA-617 [42]. In this respective analysis, PSMA-TV was also a significant negative prognosticator of OS, with PSMA-TLQ resulting an independent prognostic factor for OS. Again, Widjaja et al. [43] attempted to correlate pre-therapy PSMA PET parameters with biochemical response after two cycles of RLT. According to their results, PSA reduction correlated with the pre-therapeutic SUVmax (rho = −0.49; *p* < 0.001), but not with PSMA-TV and PSMA-TL. Moreover, multivariate analysis identified these SUVmax and age as independent predictors for early PSA response during the treatment (HR 7.94, *p =* 0.001, and HR 8.05, *p =* 0.002, respectively).

Lastly, Grubmüller et al. [44] were among the first to evaluate whether PSMA PET parameters, such as the SUVmean and total tumor volume, were associated with OS, PSA values, and RECIST response in a group of 55 mCRPC patients during RLT. Change in tumor volume was the only parameter associated with biochemical response (*p =* 0.15), while both Δ tumor volume and ΔPSA levels were significant predictors of OS (HR 1.001, *p =* 0.04, and HR 1.004, *p =* 0.01, respectively), suggesting total tumor volume as an affordable parameter for response assessment, which might overcome the limitation of RECIST in this type of patients.

Another important line of research is related to the biology of these tumors. In fact, it is well known that some patients respond to RLT and others respond poorly, probably due to individual differences in radiopharmaceutical distribution in malignant lesions, which cause variation in the adsorbed tumor dose [45]. Organs like salivary glands, the liver, and the kidneys normally exhibit PSMA expression and have recently obtained interest as benchmarks for semi-quantitative stratification of lesion uptake [46]. In fact, PSMA distribution in PET scans may anticipate radioligand binding during RLT; therefore, PSMA PET is essential for the selection of patients best suited for treatment [47]. Based on this data, the EANM procedures guideline for RLT has recently defined that a lesion uptake above the mean uptake of the liver is to be considered a prerequisite for RLT [48].

Hohberg et al. [49] obtained the absorbed dose of [^177^Lu]-PSMA in lymphatic versus osseous metastases in a cohort of 30 mCRPC patients, in order to estimate the response behavior. They found that responder patients achieved higher absorbed doses versus non-responders both for lymph nodes and bone metastases. Moreover, there was a significant difference in the ratio of tumor-to-kidney uptake between responders and non-responders, and these values correlated with the mean dose (*p* < 0.01), making it possible to use pretherapy PSMA PET to estimate the response to treatment.

Groener et al. [50] analyzed the baseline parameters derived from PSMA PET in 61 candidate patients for RLT in order to investigate their prognostic role. SUVmax, SUVmean, and TLR were significantly correlated with lesion response (rho = 0.33, *p* < 0.001; rho = 0.32, *p* < 0.001; rho = 0.31, *p* < 0.001, respectively), and ROC analysis showed a comparable value for SUVmax, SUVmean, and TLR (i.e., 0.85, 0.87 and 0.83, respectively) for predicting lesion progression. Furthermore, the Authors also evaluated the visual uptake (V-Score) for all metastases, highlighting that lesions with V-score = 0 after RLT had lower values of SUVmax, SUVmean, and TLR at baseline than those with V-score ≥ 1. In addition, a considerable number of lesions showing response to RTL (230/505, 41.8%) demonstrated a high level of PSMA expression (V-score ≥ 2) after treatment, thus maintaining targetability for additional cycles. Likewise, van der Sar et al. [51] aimed to assess the role of PSMA PET parameters in predicting early response to RLT of 237 lesions in 32 patients. SUVmax and SUVpeak were associated with PSMA PET response in the lesion-level analysis, while only SUVpeak was associated with the patient-level analysis.

More recently, peculiar research from Burgard et al. investigated “the tumor sink effect” in a cohort of 33 patients treated with RLT. Of note, they found a moderate inverse correlation between ∆SUVmean, from salivary glands and the spleen, and ∆TLP from PCa lesions. In addition, responder patients showed a higher mean SUV of salivary glands and the spleen after treatment than the baseline. These results could indicate the tumor sink effect as a potential marker for improving the efficacy/toxicity ratio of RLT [52].

## 6. Radium-223

Bone metastases are prevalent in more than 90% of mCRPC patients and are associated with pain, skeleton-related events, and mortality [53].

An option for the treatment of symptomatic bone metastases is represented by ^223^Ra-dichloride, an α-emitting radionuclide capable of inducing tumor cell death [54]. Previous studies, such as the phase 3 ALSYMPCA trial, have demonstrated that [^223^Ra]-dichloride significantly improves OS and reduces skeleton-related events and ALP [55]. Therefore, imaging tools to assess eligibility for [^223^Ra]-dichloride therapy, evaluating response assessment and guiding decision-making, are necessary. Currently, conventional imaging has some limitations: CT cannot clearly distinguish lesions from osteosclerosis, whereas flare phenomena reduces the specificity of a bone scan [56].

Recently, some studies have shown that PSMA PET has a better sensitivity and specificity for detecting nodal or visceral lesions than CT in mCRPC patients before [^223^Ra]-dichloride [57]. In support of this thesis, Bosch et al. [58] showed, in a prospective observational multicenter study with 122 patients who underwent [^223^Ra]-dichloride, that patients assessed by PSMA PET had a significantly longer median OS than those studied by CT (19.9 months vs. 12.4 months, *p =* 0.038), probably due to an under-detection of soft tissue lesions in the CT group. Moreover, the ALP and PSA response rates were significantly higher by PSMA PET than CT, indicating that molecular imaging is a reliable screening method to identify patients who will benefit from [^223^Ra]-dichloride therapy.

In addition, a prospective study by de Jong et al. evaluated the prognostic role of total tumor volume within bone, derived from PSMA PET, compared to CT and bone scan [59]. Notably, good responders had a lower skeletal tumor volume than poor responders at baseline and also after three cycles, while conventional imaging was not different between the two groups. Moreover, skeletal tumor volume was also associated with a higher risk of new extra-bone lesions.

Finally, Grubmüller et al. retrospectively assessed PSMA PET parameters (e.g., total tumor volume, SUVmean, SUVmax, and SUVpeak) in 43 patients before and after systemic therapies, also including [^223^Ra]-dichloride. All before mentioned PET parameters, as well as RECIST criteria, were significantly associated with PSA response [60].

## 7. Optimal Response Assessment with Different Criteria

In current clinical practice, therapy response assessment is traditionally based on conventional imaging, including CT and bone scintigraphy, typically performed after 12–16 week of therapy as proposed by PCWG3 guidelines.

Currently, the evaluation of imaging is based on RECIST criteria, but it is well known that its accuracy is reduced with sclerotic bone metastases, a frequent condition in mCRPC patients [61]. Moreover, conventional imaging has limited sensitivity and specificity also for small lymph nodes, especially at low PSA levels [62].

Hence, the introduction of PSMA imaging, improving the detection of PCa lesions compared to conventional scans, has encouraged a new approach for therapy response assessment as recommended in current guidelines [63]. Different evolved response criteria have been proposed, such as RECIST 1.1 or PET Response Criteria in Solid Tumors (PERCIST). More recently, Fanti et al. introduced the PSMA PET progression (PPP) criteria for a potential use in mCRPC [64], although data regarding the prognostic value of this criteria are still limited and heterogenous.

This is hereby an overview of the most updated studies comparing these criteria in relation to different types of systemic therapy (Table 1 and Table 2).

In the PSMA-PROSTATA study, Calderoni et al. [81] compared the PSMA PET response with PSA variation as prognostic factors for PFS and OS. Overall, 160 patients with CRPC were analyzed. The PSMA PET response and PSA variation showed a 79% agreement (κ = 0.553, *p* < 0.001). Moreover, the PSMA PET response was significantly associated with PFS, but not with OS, and independent from PSA variation.

On the other hand, Denis et al. [76] compared PSMA PET and conventional imaging (i.e., bone scan and CT) response evaluation to a novel hormonal agent in 18 mCRPC patients. According to the results, volumetric parameters from PSMA PET improved the response assessment as early as 4 weeks after treatment, although caution should be used because of the possibility of flare phenomenon, allowing us to improve the understanding of resistance to therapy.

Some studies analyzed the same topic in patients undergoing chemotherapy. For example, Lunger et al. [82] performed a study to assess the prognostic utility of conventional response criteria (imaging and biochemical) and PSMA PET regarding OS in a cohort of 103 patients treated with taxanes. The response was assessed by RECIST 1.1, adapted(a) PERCIST, aPCWG3, and PPP. All the PET-based criteria showed a prognostic utility, but in particular, PPP resulted in it being simple to use and more reliable in identified patients with PD. Otherwise, aPERCIST identified a subgroup of responders showing a significantly better OS. Likewise, Seitz et al. [65] performed PSMA PET in 23 patients during therapy with docetaxel. RECIST 1.1 and PERCIST criteria were correlated and compared with a biochemical response. The correlation with PSA response was higher for PET response than CT (56% vs. 33%). Moreover, for specific metastatic sites, the performance of PSMA PET seemed to be superior to that of CT.

Other studies instead focused on the identification of the best criteria to evaluate or predict the response to RLT. Gafita et al. [77] compared different response criteria, i.e., RECIST 1.1, aPCWG3, aPERCIST, PPP, and the RECIP 1.0 in 124 mCRPC patients treated with [^177^Lu]-PSMA. The response, interpreted by consensus among three blinded readers, was classified as PD vs. non-PD. Notably, the higher prognostic values and inter-reader robustness were achieved using PSMA PET-specific criteria rather than CT-based or PSMA-adapted criteria.

The same group elaborated a score for response assessment based on RECIP and PSA variation. Overall, 124 mCRPC patients treated with RLT underwent PSMA PET at baseline and after 12 weeks. RECIP 1.0 criteria were demonstrably prognostic for OS; therefore, they could potentially be used as a response biomarker for monitoring the early efficacy of [^177^Lu]-PSMA in men with mCRPC. In addition, PSA + RECIP may be used as a novel composite endpoint in mCRPC clinical trial design [78].

Furthermore, in another study, they evaluated the agreement between quantitative RECIP, a software-based analysis for calculating tumor volume, and visual RECIP by nuclear medicine physicians in order to determine response assessment in mCRPC patients treated with [^177^Lu]-PSMA. The analysis of 124 men showed an excellent agreement between visual and quantitative RECIP (κ = 0.89), as well as among readers in classifying visual RECIP PD versus non-PD (κ = 0.81). Again, RECIP PD was associated with significantly shorter OS compared to non-PD (HR 2.6, *p* < 0.001) [83].

Heinzel et al. [66] evaluated the use of PSMA PET for monitoring the response in a group of 48 patients who underwent imaging before the first and after the third or fourth treatment cycle. The response was defined according to PERCIST criteria; PSMA PET showed a sensitivity of 85% and specificity between 55 and 65%, with a negative predicted value and positive predictive value ranging between 70% and 78%, indicating that this method could be a useful tool for the evaluation of response. Likewise, Prasad et al. [70] assessed the role of interim PSMA PET, defined as a scan performed 8–10 weeks after the second cycle. Based on RECIST 1.1, 20/38 patients (52.6%) achieved PR; in 9/38 (23.7%), the disease was stable; and in 23.7%, the disease progressed with a better result in distinguishing PD from non-PD in comparison to PSA. The median OS stratified according to PET data was as follows: PR 25 months, SD 30.6 months, and PD 13.1 months (*p =* 0.013).

A study conducted by Gupta et al. [67] evaluated the different accuracy between the biochemical response, RECIST 1.1, MD Anderson (MDA) criteria for morphological response, PERCIST 1.0, and European organization for research and treatment (EORTC) criteria for molecular response. The results were as follows: the proportion of PD, PR, and SD by PSA reduction and RECIST was 9 (39.13%), 3 (13.04%), 11 (47.83%), 5 (21.74%), 2 (8.70%), and 16 (69.57%), respectively. According to PERCIST and EORTC, the proportions were 8 (34.78%), 5 (21.74%), and 10 (43.48%). Indeed, the molecular criteria (PERCIST, EORTC) resulted better than RECIST 1.1 in terms of response assessment and showed a good agreement with BR data.

Lastly, the objective of Murthy et al. [84] was to evaluate the prognostic value of end-of-treatment PSMA PET by RECIP criteria in order to define PD versus no-PD status. Overall, 10 of 20 patients (50%) had PD at the time of PET and had also a shorter OS than patients with no PD (median OS, 10.7 months vs. non-reached; *p =* 0.007), supporting the use of these criteria as a prognostic tool in mCRPC.

## 8. Synergic Role of a Dual Tracer (FDG/PSMA)

It is well known that glucose metabolism is correlated with cell proliferation and this is also valid for PCa. Suman et al. [68] have reported, in fact, that PCa with high glucose metabolism expressed by an SUVmax > 15 is associated with a Gleason score > 8 and a poorer prognosis. However, approximately 87% of mCRPC patients have at least one FDG-positive metastasis [89]. In this regard, Bauckneht et al. [71] enrolled 44 mCRPC patients undergoing FDG PET in the six months preceding systemic treatment and quantified the disease burden by means of metabolic tumor volume (MTV) and total lesion glycolysis (TLG). The results showed that a decrease in MTV value < 325.97 cm^3^ and TLG < 844.86 was associated with increased OS, highlighting the importance of FDG evaluation in this type of patient.

Similarly, in a retrospective study, Güzel et al. [85] aimed to evaluate the prognostic role of volumetric parameters obtained from PSMA-PET and FDG-PET in 71 mCRPC patients receiving taxane therapy after imaging. FDG and PSMA total tumor volume (TTV-F, TTV-P), total lesion glycolysis (TTL-G), and total lesion PSMA (TTL-P) values were calculated and the correlation of these parameters with OS was investigated. According to the analyses, all the above-mentioned parameters, as well as their combination, were found to be prognostic factors in predicting shorter OS. Moreover, on multivariate analysis, TTL-G + P was found to be an independent prognostic factor to OS.

Regarding the same topic PSMA/FDG, the Australian and New Zealand Urogenital and Prostate Cancer Trial Group prospectively collected the quantitative parameters of both pre-treatment PET in the TheraP cohort (Lu-PSMA-617 vs. cabazitaxel), measuring MTV, SUVmax, and SUVmean. The odds of response to RLT vs. chemotherapy were markedly higher for patients with PSMA-PET SUVmean ≥ 10 (OR 12.2 vs. 2.2 *p =* 0.039). Moreover, a higher FDG-PET MTV (≥200 mL; OR 0.44; *p =* 0.035) was associated with a worse response regardless of treatment [79].

A sufficient PSMA expression is, therefore, necessary for the success of RLT and is verified by PSMA PET. However, some patients do not demonstrate an acceptable response, and others, initially with a good response, experience a progressive disease probably due to disease heterogeneity [90]. To overcome this problem, an additional analysis by an FDG scan seems to be a suitable method because, in advanced mCRPC, the glucose metabolism can be increased in parallel with the missing PSMA expression [91]. As a matter of fact, in a study involving 29 patients with worsening disease after a median of 4 RLT cycles, FDG and PSMA PET were combined in order to evaluate the prognostic implication of mismatch findings. In particular, 17/29 (59%) of patients showed at least one mismatch metastasis, and of them, the median OS, was significantly shorter than those without (3.3 vs. 6.1 months, *p =* 0.008); subjects with a high MTV by FDG obtained a shorter OS of 2.6 months than patients with low MTV (5.3 months, *p =* 0.034). There was also a trend to short OS in patients with high SUVmax and high TLG obtained by FDG scan; put together, these results demonstrate the importance of combined FDG and PSMA PET for the identification of different phenotypes of disease that could require a change in therapy management [72].

On the other hand, Pathmanandavel et al. [86] developed a prospective study (NOX66-LuPIN) aiming to determine the prognostic value of posttreatment TTV and SUV on both PSMA and FDG PET. Fifty-six men were analyzed with the following results: an increase in PSMA TTV by at least 30% was linked with worse OS (median, 10.2 vs. 23.6 months; *p =* 0.002), while an FDG TTV increase by more than 30% was associated with a short PFS (median, 3.5 vs. 8.6 months; *p* < 0.001), but not OS. On multivariate analysis, only increased PSMA TTV and PSA values remained independently prognostic of OS (HR 5.1, *p =* 0.008, and HR 3.5, *p =* 0.03, respectively. Moreover, Ferdinandus et al. [69] performed another single-center prospective study with the intent of identifying the prognostic value of baseline imaging in patients enrolled in the LuPSMA trial. In addition to conventional blood markers, such as ALP (HR 1.1; 95% CI, 1–1.2) and LDH (HR 1.2; 95% CI, 1–1.5), FDG-positive tumor volume (HR2.6; 95% CI, 1.4–4.8) and mean intensity of PSMA-avid metastases uptake (HR 0.89; 95% CI, 0.8–0.98) also demonstrated a prognostic significance for survival in mCRPC undergoing RLT, confirming that patients with low volumes of FGD-avid disease had a longer OS than other patients (6.1 vs. 9.6 months, *p* < 0.001).

A bi-centric study by Michalski et al. [73] tried to investigate the prognostic value of combined PSMA PET and FDG PET regarding outcome prediction in a cohort of 54 patients. They compared patients with at least one FDG-positive but PSMA-negative lesion (FDG+/PSMA−) versus patients without FDG+/PSMA−. In one-third of the cases, patients presented with FDG+/PSMA− lesions (33%), mostly in the skeleton or liver in 44% of the cases. A significantly lower OS was found in the first group (*p* < 0.001) with a median survival of 6.0 ± 0.5 months compared to the other (16.0 ± 2.5 months). These results probably highlight, once again, a more aggressive biology of the disease in FDG-positive patients, which should be studied in depth if this population still benefits from RLT.

In the end, Telli et al. [87] evaluated OS and PFS in a prospective study including 52 patients, who received two to six cycles of RLT. A comprehensive assessment of tumor burden has been performed and compared with the PSA response rate. PSA doubling time ≤ 2.4 months (HR 15.7, *p* < 0.0001) and primary resistance (HR 4.9, *p =* 0.003) were the most significant parameters related to poor OS; moreover, initial FDG-positive disease (HR 0.59, *p =* 0.03) and a <30% PSA response rate after first RLT administration (HR 1.016, *p =* 0.003) were correlated with an unfavorable survival. Regarding PFS, the presence of liver metastases, FDG-positive disease, a short PSA doubling time, and a high TLG/PSMA-TL were associated with shorter outcomes.

## 9. Future Perspectives

In addition to the therapies considered the standard of care today, some studies are exploring the potential role of further options for mCRPC patients, with particular regard to nuclear medicine compounds. Recently, PSMA-targeting alpha-particle RLT with [^225^Ac]-PSMA-617 was proposed as a new treatment approach in these patients after the end of all available standard therapy. A promising anti-tumor distribution was observed, therefore resulting in a new option [92,93]. On the basis of data obtained from PSMA PET for other systemic therapies, Unterrainer et al. [80] have investigated the role of TTV in a cohort of patients treated with [^225^Ac]-PSMA-617. They observed that there was a significant decrease in TTV after two cycles compared to the baseline (median 835 vs. 2021 mL; *p =* 0.028), with an associated decline in PSA value (median 178 vs. 686.6 ng/dL, *p =* 0.018). Moreover, the TTV value was superimposable between the short and long survival, allowing the possibility that even patients with an important tumor load might profit from this type of RLT. Similarly, Rosar et al. [74] investigated the prognostic value of TLP and MTV in 17 patients undergoing tandem therapy (Ac-PSMA after progression with Lu-PSMA). Both therapies were well tolerated without acute adverse events. PET-derived parameters and biochemical markers were mostly concordant and the difference in OS was significant for the PET response assessment (median OS not reached vs. 8.3 m, *p =* 0.044), but not for the PSA response assessment (median OS 18.1 months vs. 9.4 months, *p =* 0.468); so, molecular imaging appears to be superior to conventional biomarkers in estimating survival outcomes. Despite these interesting data, there is currently no accurate decision addressing the optimal sequence for RLT, but many clinical trials are underway with β and α emitters tested alone, together, or in combination with other therapies in various PCa stages [94].

This amount of data provided so far makes it necessary to develop new tools to know their actual usefulness. Recently, Karpinski et al. [88] aimed to combine reproducible PSMA-PET metrics into nomograms or accurate predictions of pan-stage OS. They collected imaging and clinical and follow-up data from a large cohort (667 patients) to compare PSMA PET with established clinical risk scores. PSMA PET scans were performed following international consensus guidelines and analyzed in accordance with PROMISE (Prostate Cancer Molecular Imaging Standardized Evaluation) criteria to obtain the molecular imaging tumor–node–metastases status, total tumor lesion count, total tumor volume, and total tumor expression (SUVmean). In the combined development and internal validation cohort, the quantitative PPP nomogram was superior to conventional scores, such as the STARCAP or EAU score, accurately stratifying high-risk and low-risk groups for OS in both early and late stages of PCa. Consequently, in the future, the combination of clinical and imaging findings might yield a superior prognostic value.

Finally, interesting results are expected in the coming years with the use of radiomics and artificial intelligence, as well as in mCRPC. With regard to this topic, Roll et al. [75] analyzed the predictive and prognostic value of radiomic features from PSMA PET/RM in 21 mCRPC patients before RLT. Ten independent features (three PET-, one T2-, and six T1-post-gadolinium-derived features) were able to well differentiate responders from non-responders. However, only the interquartile range T2-derived feature showed the highest accuracy (AUC = 0.83) for the prediction of PSA response after RLT. Moreover, the same parameter as well as biochemical response were independent prognostic factors for OS (*p =* 0.038 and *p =* 0.003, respectively). These data confirm the potential use of radiomic analysis from the baseline PSMA PET before RLT for a more tailored therapy.

## 10. Conclusions

To summarize, mCRPC remains a challenge in clinical oncology, although the number of new therapeutic agents is steadily increasing. PSMA PET imaging offers a higher sensitivity and specificity than conventional imaging and provides accurate staging and efficient diagnosis of distant metastases. The data presented herein highlight the usefulness of PET in risk stratification, with a prognostic potential that can have a significant impact on clinical practice. Some limitations of our overview must be addressed: firstly, the retrospective nature of most studies and moreover, the heterogeneity of the therapies analyzed. Despite this, several prospective trials are ongoing and will probably provide more evidence supporting the prognostic potential of PET PSMA data in mCRPC.

## Figures and Tables

**Table 1:** Outcome results of PSMA PET prognostic role in the different therapeutic regimens in mCRPC.

Author	Year	Design	Cohort	Treatment	Outcomes
Seitz et al. [65]	2018	R	7 mCSPC 16 mCRPC	DTX	PSMA PET seems superior to convetional CT for response assessment
Plouznikof et al. [23]	2019	R	11 15	enzalutamide AA	PSMA expression changes are associated with response
Grubmüller et al. [44]		R	38	[^177^Lu]-PSMA	ΔTTV was associated with OS
Heinzel et al. [66]		R	48	[^177^Lu]-PSMA	PSMA PET could be a useful tool for RLT assessment
Gupta et al. [67]		NR	46	[^177^Lu]-PSMA	Molecular criteria were superior to morphological ones
Suman et al. [68]		R	40	[^177^Lu]-PSMA	the higher FDG uptake was associated with high GS and shorter PFS
Grubmüller et al. [60]	2020	R	9 12 22 6 18	[^223^Ra] cabacitaxel DTX AA enzalutamide	PSMA PET parameters were able to predict response to systemic therapies
Ferdinandus et al. [69]		R	50	[^177^Lu]-PSMA	PSMA and FDG PET parameters were prognostic factors of OS
Markowski et al. [21]	2021	P	6	BAT	^18^F-DCFPyL can indicate early progression
Karyağar et al. [22]		R	34	enzalutamide	PSMA-TV predict PSA response
Has Simsek et al. [32]		R	52	DTX	High PSMA-TV was associated with shorter OS and DTX failure
Seifert et al. [41]		R	33	[^177^Lu]-PSMA	PSMA-TV was a prognostic for OS only excluding patients with low PSMA expression
Seifert et al. [42]		R	110	[^177^Lu]-PSMA	PSMA-TLQ was an independent prognosticator for OS
Widjaja et al. [43]		R	71	[^177^Lu]-PSMA	SUVmax was early predictor of PSA response
Prasad et al. [70]		R	38	[^177^Lu]-PSMA	Interim PSMA PET was predictive of OS and progression disease
Bauckneth et al. [71]		R	24 20	DTX AA or enzalutamide	TLG was associated with PFS
Khreish et al. [72]		R	29	[^177^Lu]-PSMA	Combination of FDG and PSMA PET was a significant prognostic factor of OS
Michalski et al. [73]		R	54	[^177^Lu]-PSMA	FDG+/PSMA− lesions are a negative predictor of OS
Rosar et al. [74]		R	17	[^225^Ac]+ [^177^Lu]-PSMA	Molecular imaging response appears to be superior to PSA change in estimating survival outcome
Roll et al. [75]		R	21	[^177^Lu]-PSMA	Radiomics analysis may offer new predictive and prognostic parameters
Telli et al. [34]	2022	R	32 22	DTX enzalutamide/AA	PSMA-TV was associated with OS
Rosar et al. [39]		P	66	[^177^Lu]-PSMA	Early ΔTLP predicted OS
Denis et al. [76]		R	18	NHA	Volumetric parameters can improve response evaluation; early response need to be approached with caution because of flare
van der Sar et al. [51]		R	32	[^177^Lu]-PSMA	SUVpeak of the most avid lesion was predictive of response to RLT
Gafita et al. [77]		R	124	[^177^Lu]-PSMA	PSMA criteria showed higher prognostic values
Gafita et al. [78]		R	124	[^177^Lu]-PSMA	RECIP 1.0 is prognostic for OS
Buteau et al. [79]		P	101 99	cabacitaxel [^177^Lu]-PSMA	PSMA-PET SUVmean was predictive of favourable response to RLT than chemotherapy; High MTV was associated with scarce response to both treatments
Unterrainer et al. [80]		R	13	[^225^Ac]-PSMA	ΔTTV seems to improve the response assessment
Ke et al. [20]	2023	R	106	AA	Low PSMA-TV, low TLR were associated with biochemical response and longer PFS
Shagera et al. [24]		R	16 14	enzalutamide AA	Both PSMA response criteria are associated with OS
Shagera et al. [33]		R	32	cabacitaxel	PSMA-TV associated with PFS and OS
Bugard et al. [52]		R	33	[^177^Lu]-PSMA	Tumor sink effects were observed in the salivary glands and spleen
de Jong et al. [59]		P	28	[^223^Ra]	Lower TTV_bone_ was associaterd with best clinical outcome
Calderoni et al. [81]		R	160	elegible for second- or subsequent-line therapy	PSMA PET parameters were able to predict response and associated with PFS
Lunger et al. [82]		R	103	Taxane-based	PPP was the best prognostic criteria for OS
Gafita et al. [83]		R	124	[^177^Lu]-PSMA	visual RECIP was not inferior to quantitative RECIP
Murthy et al. [84]		R	20	[^177^Lu]-PSMA	PSMA PET at the end of treatment was prognostic for OS
Guzel et al. [85]		R	71	Taxane-based	PSMA and FDG PET parameters had a prognostic value
Pathmanandavel et al. [86]		P	37	[^177^Lu]-PSMA	PSMA TTV was prognostic of OS
Telli et al. [87]		P	52	[^177^Lu]-PSMA	FDG > PSMA, high MTV, TLG/TL-PSMA were associated with poor OS
Bosch et al. [58]	2024	P	122	[^223^Ra]	PSMA PET seems to be a valuable tool for identifying patients who will benefit from ^223^Ra therapy
Karpinski et al. [88]		R	667	different systemic therapies	PPP nomograms accurately stratify high-risk and low-risk groups for OS

Abbreviations: AA, abiraterone acetate; BAT, bipolar androgen therapy; DTX, docetaxel; FDG, fluorodeoxyglucose; MTV, metabolic tumor volume; OS, overall survival; PFS, progression-free survival; PPP, PSMA PET progression criteria; PSMA, prostate-specific membrane antigen; RECIP, response evaluation criteria in prostate-specific membrane antigen (PSMA) PET/CT; RLT, radioligand therapy; SUVs, standardized uptake values; TL, total lesion; TLG, total lesion glycolysis; TLP, total lesion PSMA; TV tumor volume; TTV, total tumor volume; TLR, tumor-to-liver ratio.

**Table 2:** Principal PET parameters and response criteria used for assessment in mCRPC.

Author	Radiotracer	PET Evaluation	PET Metrics	Response Criteria
Seitz et al. [65]	[^68^Ga]-PSMA-11	Baseline, after 3 or 6 cycles	-	PSA, PERCIST, RECIST 1.1
Plouznikof et al. [23]	[^68^Ga]-PSMA	Baseline, no more than 1 y later	SUVmax	Visual, RECIST 1.1, PSA
Grubmüller et al. [44]	[^68^Ga]-PSMA-11	Baseline, after 3 cycles	SUVmean, TTV	mPERCIST, RECIST 1,1
Heinzel et al. [66]	[^68^Ga]-PSMA-11	Baseline, after 3 or 4 cycles	∆SUVmean, ∆ highest SUV	mPERCIST, PCWG3
Gupta et al. [67]	[^68^Ga]-PSMA-11	Baseline, after 8–12 weeks	SUVmax	PCWG3, RECIST 1.1, PERCIST, EORTC, MDA
Suman et al. [68]	[^68^Ga]-PSMA-11 [^18^F]-FDG	At 10–12 weeks	-	ECOG/KarnofskyHRQoL scores, PCWG3, PERCIST, RECIST 1.1
Grubmüller et al. [60]	[^68^Ga]-PSMA-11	Baseline, within 6 weeks after therapy	SUVmax, SUVmean, SUVpeak, TTV, and their ∆ values	PCWG3, mPERCIST
Ferdinandus et al. [69]	[^68^Ga]-PSMA-11 [^18^F]-FDG	Baseline	SUVmax, SUVmean, TMV	-
Markowski et al. [21]	[^18^F]-DCFPyL	Baseline, after 3 months	SUVmax	Visual, RECIST 1.1., PCWG3
Karyağar et al. [22]	[^68^Ga]-PSMA-11	Baseline	SUVmax, SUVmean, PSMA-TV, PSMA-TL	PSA
Has Simsek et al. [32]	[^68^Ga]-PSMA-617	Baseline	SUVmax, SUVmean, PSMA-TV, PSMA-TL	PSA
Seifert et al. [41]	[^68^Ga]-PSMA-11	Baseline, interim	PSMA-TV, TLQ	mPERCIST, RECIST
Seifert et al. [42]	[^68^Ga]-PSMA-11	Baseline	PSMA-TV, PSMA-TLQ, PSMA-TLU	-
Widjaja et al. [43]	[^68^Ga]-PSMA-11	Baseline	Average SUVmax, PSMA-TV, PSMA-TL	PCWG2
Prasad et al. [70]	[^68^Ga]-PSMA	Baseline, after 2 cycles	-	RLT-REC-PCA, PCWG3
Bauckneht et al. [71]	[^18^F]-FDG	6 months before	SUVmax, MTV, TLG	-
Khreish et al. [72]	[^68^Ga]-PSMA-11 [^18^F]-FDG	at the time point of worsening disease	SUVmax, MTV, TLG of mismatched lesions	-
Michalski et al. [73]	[^68^Ga]-PSMA [^18^F]-FDG	Baseline	visual	-
Rosar et al. [74]	[^68^Ga]-PSMA-11	Baseline, after 1 cycle	TLP, MTV	PCWG3, mPERCIST
Roll et al. [75]	[^68^Ga]-PSMA-11 PET/MRI	Baseline	SUVmean, SUVmax, SUVmedian, TTV, 378 radiomics features	-
Telli et al. [34]	[^68^Ga]-PSMA	Baseline	SUVmax, SUVmean, PSMA-TV, PSMA-TL	PSA
Rosar et al. [39]	[^68^Ga]-PSMA-11	Baseline, after 2 cycles	TLP, ΔTLP	PERCIST, PCWG3
Denis et al. [76]	[^68^Ga]-PSMA-11	Baseline, after 4 and 12 weeks	SUVmax, SUVmean, PSMA-TV, PSMA-TL	EAU/EANM
van der Sar et al. [51]	[^68^Ga]-PSMA-11	Baseline, after 2 cycles	SUVpeak, SUVmean, PSMA-TV, PSMA-TL	PERCIST
Gafita et al. [77]	NR	Baseline, at 12 weeks	-	RECIST 1.1, aPCWG3, aPERCIST, PPP, and RECIP 1.0
Gafita et al. [78]	NR	Baseline, at 12 weeks	-	RECIP 1.0, PSA+RECIP
Buteau et al. [79]	[^68^Ga]-PSMA [^18^F]-FDG	Baseline	SUVmean, MTV	-
Unterrain et al. [80]	[^18^F]-PSMA-1007	Baseline, after 2 cycles	TTV, ∆TTV, ∆SUV_mean_TTV_, ∆SUV_max_TTV_	mPERCIST, RECIST 1.1
Ke et al. [20]	[^68^Ga]-PSMA-11	Baseline	SUVmax, SUVmean, SUVpeak, PSMA-TV, TLR	PSA
Shagera et al. [24]	[^68^Ga]-PSMA	Baseline, after 12 ± 4 week	PSMA-TV	EAU/EANM, RECIP 1.0
Shagera et al. [33]	[^68^Ga]-PSMA-11	Baseline	SUVmax, SUVmean, PSMA-TV, PSMA-TL	-
Bugard et al. [52]	[^68^Ga]-PSMA-11	Baseline, after 2 cycles	TLP, ΔTLP	PERCIST, RECIP 1.0, PCWG3
de Jong et al. [59]	[^68^Ga]-PSMA	Baseline, after 3 and 6 cycles	Visual, TTV_bone_	PERCIST
Calderoni et al. [81]	[^68^Ga]-PSMA-11	Baseline, interim	SUVmax, tumor burden	PSMA PET/CT consensus statement
Lunger et al. [82]	[^68^Ga]-PSMA-11	Baseline, up to 3 months	-	PPP, aPERCIST, aPCWG3
Gafita et al. 83]	NR	Baseline, at 12 weeks	-	visual RECIP, quantitative RECIP
Murthy et al. [84]	[^68^Ga]-PSMA-11	Baseline, after last cycle	PSMA-VOL	PSMA VOL >20%, RECIP 1.0, PCWG3
Guzel et al. [85]	[^68^Ga]-PSMA [^18^F]-FDG	Baseline	Visual, TV-F, TV-P, TTV-F, TTV-P, TL-G, TL-P, TTL-G, TTL-P, TTV-F +P, TTL-G+P	-
Pathmanandavel et al. [86]	[^68^Ga]-PSMA-11 [^18^F]-FDG	Baseline, after treatment	SUVmax, SUVmean, TTV	-
Telli et al. [87]	[^68^Ga]-PSMA, [^18^F]-FDG	Baseline	SUVmax, PSMA-TV, PSMA-TL, MTV, TLG	PCWG3
Bosch et al. [58]	[^68^Ga]-PSMA-11 [^18^F]-PSMA-1007	Baseline, after 4–8 weeks	STI, bPSMA, pPSMA	ALP, PSA, ALSYMPCA trial
Karpinski et al. [88]	[^68^Ga]-PSMA-11, [^18^F]-PSMA-1007	Baseline	TTV, SUVmean	-

Abbreviations: ALP, alkaline phosphatase; a-, m- PERCIST, adapted, modified PET Response Criteria in Solid Tumors FDG, fluorodeoxyglucose; EORTC, European Organization for Research and Treatment of Cancer; MDA, MD Anderson Cancer Center criteria; MTV, metabolic tumor volume; PCWG, prostate cancer working group; PSMA, prostate-specific membrane antigen; RECIP, response evaluation criteria in prostate-specific membrane antigen (PSMA) PET/CT; bPSMA, baseline PSMA; pPSMA, post-treatment PSMA; RECIST, Response Evaluation Criteria in Solid Tumors; RLT-REC-PCA, RadioLigand Therapy Response Evaluation Criteria for Prostate Cancer; SUVs, standardized uptake values; STI, soft-tissue involvement; TL, total lesion; TLG, total lesion glycolysis; TLP, total lesion PSMA; TV, tumor volume; TMV, total molecular volume; TTV, total tumor volume; TLR, tumor-to-liver ratio. Notes: ALSYMPCA trial [80].

## Data Availability

The data presented in this study are available on request from the corresponding author.

## Abbreviations

AA	abiraterone acetate
ADT	androgen deprivation therapy
BAT	bipolar androgen therapy
EANM	European Association of Nuclear Medicine
EAU	European Association of Urology
EORTC	European organization for research and treatment
mCRPC	metastatic castration-resistant prostate cancer
MTV	metabolic tumor volume
Pca	Prostate Cancer
PCWG3	Prostate Cancer Working Group 3
PERCIST	Response Criteria in Solid Tumors
RECIP	Response Evaluation Criteria in PSMA PET/CT
RECIST	Response Evaluation Criteria in Solid Tumors
RLT	radioligand therapy
TL	total lesion
TLG	total lesion glycolysis
TLP	total lesion PSMA
TLQ	total lesion quotient
TLR	tumor-to-liver ratio
TLU	total lesion uptake
TMV	total molecular volume
TTV	total tumor volume
TV	total volume
